# Enhanced healing of oral chemical burn by inhibiting inflammatory factors with an oral administration of shengFu oil

**DOI:** 10.3389/fphar.2022.913098

**Published:** 2022-08-11

**Authors:** Xin Yin, Jing Hong, He-Bin Tang, Min Liu, Yu-Sang Li

**Affiliations:** ^1^ Lab of Hepatopharmacology and Ethnopharmacology, School of Pharmaceutical Sciences, South-Central Minzu University, Wuhan, China; ^2^ Chongqing Center for Drug Evaluation and Inspection, Chongqing, China

**Keywords:** Shengfu oil, Frankincense, oral chemical burn, subacute toxicity, inflammatory microenvironment

## Abstract

ShengFu oil is a compounded Chinese medicinal prescription, and provides antibacterial, anti-inflammatory, and analgesic effects, favoring burn wound repair. In this study, we aimed at investigating the effects of topical applications of ShengFu oil and its active ingredients in oral chemical burns and elucidating its regulatory effects on β-catenin, COX-2, and MMP-9 expression caused by exposure to acid or alkaline agents. ShengFu oil contains 16 components, such as Frankincense, Radix Scutellariae and Radix Rehmanniae, and the main active ingredients from Frankincense are α-pinene, linalool, and n-octanol. Mouse models of oral chemical burns were induced by using glacial acetic acid or sodium hydroxide. Hematoxylin and eosin staining and immunohistochemical staining were used to detect the protein expressions of β-catenin, COX-2, and MMP-9 in wound tissues. They were further quantified by multispectral imaging analysis to clarify the effective mechanism of ShengFu oil for intervening inflammatory factors and active components. Our results indicated that the application of ShengFu oil on oral chemical burns effectively stopped the oral burn bleeding and reduced the inflammatory reaction in the damaged tissues, demonstrating that ShengFu oil can promote wound tissue repair in burns caused by heat, acids, and alkalis. The immunohistochemical staining results illustrated that ShengFu oil and its active ingredients significantly reversed the abnormal changes in inflammation-related proteins in mouse tongue tissues that were caused by chemical burns. Regarding long-term toxic effects of ShengFu oil on the gastrointestinal tract, liver, and kidney system, the results of hematoxylin and eosin staining experiments depicted that ShengFu oil was safe and effective for liver, kidney, intestine, esophagus, and tongue. All of these demonstrated that ShengFu oil and its active ingredients are effective and safe in preventing and treating oral chemical burns by interfering with the inflammatory microenvironment.

## Introduction

Oral cavity can serve as a protective barrier against the external environment (Liu et al., 2010; Gaffen and Moutsopoulos, 2020). Once this barrier is disrupted, tissue damage and microbial dysbiosis are triggered, leading to diseases such as periodontitis, oral mucosal disease, and oral cancer (Dutzan et al., 2017). Oral chemical burns occur when a corrosive chemical is ingested intentionally or inadvertently, causing burns and ulcers in the oral mucosa, esophagus, stomach, and upper digestive tract (Chirica et al., 2017). If the treatment is delayed or incomplete, chronic inflammation of the wounds occurs and may even lead to oral cancer (Patel et al., 2011). Therefore, coordinated wound healing is essential to restore the interrupted anatomical continuity and functional states of the mucosa. Wound healing is a complicated and multifactorial process that leads to wound contraction and closure and restoration of functional barriers (Singh et al., 2006). The current therapeutic strategies for oral chemical burn healing, including local application of corticosteroids such as dexamethasone and use of antimicrobials, need to be replaced by better alternatives due to their high cost, appearance of dissatisfactory side effects, and overuse of antibiotics leading to increasing antibiotic resistance (Oryan et al., 2017). Additionally, surgical debridement is required to remove necrotic tissue and accelerate the formation of granulation tissue (Wormald et al., 2020). With increasing number of plant-based traditional medicines having been proved to be safe and effective for wound healing, there is a growing demand for such inexpensive treatment modalities of burns in developing countries because they can diminish the substantial financial burden on health-care systems (Albertyn et al., 2015; Lee et al., 2015; Fana et al., 2021). Traditional medicines in such as Frankincense, Coptis Chinensis, Cortex Phellodendri, Radix Astragali, and Flos Lonicerae, which are also the components of ShengFu oil, have been proved to effectively facilitate wound healing (Fujii et al., 2017).

ShengFu oil (SFO) is a compounded Chinese medicinal prescription having analgesic, anti-inflammatory, and saprophytic muscle effects. It is prepared by modern extraction technology using 16 traditional medicines such as Frankincense, Scutellariae radix, and Rehmanniae radix (Jia et al., 2013; Chen et al., 2017). Many previous studies have reported that Frankincense, a component of ShengFu oil, exhibits significantly anti-inflammatory and analgesic effects because of its active ingredients such as α-pinene, linalool, and n-octanol (Li et al., 2016). Clinical observations in the treatment of 80 cases of first- or second-degree burn revealed that the pain gradually disappeared, and the wound was not reinfected. Additionally, no obvious scar appeared, and new hair grew after the treatment. ShengFu oil has been applied as a promising potential therapeutic agent for the treatment of full-thickness scalded skin by regulating the expression of β-catenin and COX-2 (Chen et al., 2017).

However, clinically effective treatment options for severe oral chemical burns remain unavailable. Furthermore, there are only a few published reports describing protein expression of inflammatory cytokines from oral cavity. Therefore, in this study, we aimed to evaluate different treatment modalities for oral chemical burns, and try to elucidate their regulatory effects on β-catenin, COX-2, and MMP-9.

## Materials and methods

### Materials

The following drugs were used: α-pinene (Cat. No. GF01-GNCS; TCI, Shanghai); linalool (Cat. No. H2020186; Aladdin, Shanghai, China); 1-octanol (Cat. No. 20140312; Sinopharm Chemical Reagent Co.,Ltd); sesame oil (Blessing Mill, Wuhan, China); Tween-80 (Cat. No. 0442; Amresco, United States). All the other reagents in the experiment were pure grade for analysis. The primary antibodies used in this experiment included antibodies against COX-2 (Cat. No. ab23672; Abcam Inc., Cambridge, MA, United States), β-catenin (Cat. No. ab6301; Abcam Inc., Cambridge, MA, United States), MMP-9 (Cat. No. SAB5200294; Sigma-Aldrich, United States). Chinese herbal medicines [bark of *Phellodendron amurense* Rupr. (Phellodendri cortex), rhizome of *Rheum officinale* Baill. (Rhei rhizoma), stem of *Astragalus membranaceus* (Fisch.) Bge. var. mongholicus (Bge.) Hsiao (Astragali radix), rhizome of *Coptis chinensis* Franch. (Coptidis rhizoma), root of *Scutellaria baicalensis* Georgi (Scutellariae radix), root of *Angelica dahurica* (Fisch.ex Hoffm.) Benth. et Hook. f. var. formosana (Boiss.) Shan et Yuan (Angelicae dahuricae radix), gum resin of *Boswellia carterii* (Frankincense), root of *Sanguisorba officinalis* L. (Sanguisorbae radix), root of *Lithospermum erythrorhizon* Sieb. et Zucc (Lithospermi radix), resin of *Commiphora myrrha* Eng1 (Myrrh), root of *Rehmannia glutinosa* (Gaert.) Libosch. ex Fisch. et Mey (Rehmanniae radix), rhizome of *Bletilla striata* (Thunb.) Reichb. f. (Bletillae rhizoma), root of *Cynanchum* atratum Bge (Cynanchi atrati radix), root of *Angelica sinensis* (Oliv.) Diels (Angelicae sinensis radix), extracts of stem and leaf of *Cinnamomum camphora* (L.) Presl (Camphora)] were purchased from the Yinpian factory, Guangzhou Medicine Company (Guangzhou, China). ShengFu oil and Frankincense oil extracts used in the present study were taken from the samples reported in our previous studies, which have been deposited at the sampling room of the Lab of Hepatopharmacology and Ethnopharmacology, School of Pharmaceutical Sciences, South-Central Minzu University [the same batch number: Nos. 20150317SFO for ShengFu oil (Chen et al., 2017); Nos. 20150313FOE for Frankincense oil extracts (Li et al., 2016)].

### Animal care

Male Kunming mice (18–25 g, 6 weeks) were supported by the Laboratory Animal Center, Hubei. The mice were kept for 7 days in a specific pathogen-free environment with adequate water and food, thermoregulatory conditions (22–25°C), and a 12-h light-dark cycle. In this study, all animals were raised and manipulated following protocols approved by the Animal Experimentation Ethics Committee of the South-Central Minzu University, Wuhan, China (approval number: 2019-SCUEC-AEC-002).

### Animal model establishment and administration grouping

The oral burn models were established to study the repairing effect of ShengFu oil using boiling water, glacial acetic acid, and sodium hydroxide. In the three burning models, all mice were first anesthetized intraperitoneally with 600 µl of 10% chloral hydrate (0.1 g/ml). The oral thermal burn model was established by oral administration of boiling water (500 µl) at 100°C. For the oral acid burn, a 2 mm diameter filter paper wad soaked in glacial acetic acid was applied to the dorsal surface of the mouse tongue for 25 s. The oral alkali burn was induced in the lower lip mucosa of the mouse by the application of sodium hydroxide (solid white crystals; contact surface 4 mm^2^) for 6 s, followed by rinsing the site with sterile normal saline solution until forming an ulcer. After establishing experimental animal models of oral burns, mice were randomly divided into five groups (*n* = 5 mice per group): the control (sterile normal saline solution; 15 ml/kg/d), model, ShengFu oil (15 ml/kg/d, 200 mg/ml), dexamethasone (15 ml/kg/d, 20 mg/ml), and sesame oil (15 ml/kg/d, 15 ml/kg/d) groups. Meanwhile, a total of 25 mice for alkali burns were randomly divided into five groups (*n* = 5 mice per group): the control, model, dexamethasone, low- and high-dose Frankincense groups. The low- and high-dose Frankincense groups were treated with oral administration of Frankincense oil extracts (75 and 150 mg/ml) at a dose of 15 ml/kg, respectively. The dexamethasone group was treated with dexamethasone (dexamethasone solution was prepared at a concentration of 20 mg/ml in water and then topically instilled in mice at a dose of 15 ml/kg/d), and the other groups were treated with the equal volume of sterile normal saline solution. In addition, another group of mice with alkali burn models were randomly divided into six groups: control (sterile normal saline solution: 15 ml/kg/d), model, α-pinene (0.072 mg/ml, 15 ml/kg/d), linalool (0.038 mg/ml, 15 ml/kg), n-octanol (0.059 mg/ml, 15 ml/kg/d), and combination (0.169 mg/ml, 15 ml/kg/d; α-pinene, linalool, and n-octanol mixed at 1:1:1) groups. The dosage to treat mice was calculated from the human dose using the following formula: the body surface area: Human dose (ml/kg) to mouse dose (ml/kg)—multiply by 12.3. To compare the oral repairing effects on mice, all groups, except the model group, were topically administered the corresponding drug concentrations to fully cover the wound, immediately after the burn induction for three (boiling water- and alkali-burned mice) or seven (acid-burned mice) consecutive days once a day.

### Histopathological analysis of the tongue tissues

After sacrifice, the animals’ tongues were incised in front of the peripheral papilla for macroscopic morphometric evaluation of wound healing; the fresh tissues were instantly placed in 10% neutral formalin and then embedded in paraffin. The blocks were cut into 3-µm-thick slices, fixed on microscopic slices, and stained with hematoxylin and eosin (H&E; Li et al., 2016). Histopathologic examinations of the tongue slices were conducted using a Nikon 50i light microscope (Nikon Inc., Tokyo, Japan). The inflammation score statistics were obtained from the H&E-stained sections. Scoring systems were established for chemically induced burn wounds, and inflammatory cell infiltrates were defined as the main category with respective subordinate specific criteria. The inflammatory infiltrate was quantified based on the number of inflammatory points (0 = none; 1 = 1–5 inflammatory points, mild inflammation; 2 = 6–15 inflammatory points, moderate inflammation; and 3 = > 15 inflammatory points, severe inflammation) to evaluate the severity and extent of inflammation.

### Immunohistochemical of the tongue tissues

Post deparaffinization and hydration, the paraffin-embedded tongue tissue sections’ antigens were retrieved by citric acid buffer (pH = 6.0) microwave-antigen retrieval, and the endogenous peroxidase was blocked with a 3% H_2_O_2_. Subsequently, the sections were blocked with 5% bovine-serum albumin, followed by incubation with diluted primary antibodies against COX-2 (1:200), β-catenin (1:200), MMP-9 (1:200), and secondary antibodies. After color development with 3,3′-diaminobenzidine tetrahydrochloride, the sections were counterstained with hematoxylin and mounted in permanent aqueous mounting media under coverslips. Finally, the spectral optical density of images was acquired from 420 to 720 nm in 10 nm increments with a CRi Nuance Multispectral Imaging System [Cambridge Research and Instrumentation Inc., Woburn, MA, United States (Li et al., 2012)]. Each image was spectrally unmixed by Nuance software (v3.0.2) and pure spectral libraries of individual chromogens (DAB and hematoxylin). Optical density of each marker of co-localization in DAB image represented the total expression of relative protein-positive regions. The protein expression level in the nucleus was represented by the total signal (optical density) of the co-localization marker in DAB image and the hematoxylin image. The cytoplasmic expression was obtained by comparing the difference between the expression level of DAB channel and hematoxylin channel proteins. Three same-size areas were randomly selected, and the expression of the corresponding protein in each image minus the measured background noise was quantified.

### Repeated dose 30-days oral toxicity study

Ten mice were chosen for this study after a period of acclimation and quarantine, the 30-days repeated-dose oral study. The animals were randomly assigned to two groups of five animals each, and treated with the SFO at a dose of 300 µl or saline by oral gavage twice daily for 30 days. All mice were observed for mortality, behavioral changes, or sign of any toxicity once a day during the study period. At the end of the experiment, blood sampling was collected by harvesting the mice eyeballs; further, the levels of aspartate aminotransferase (AST), alanine aminotransferase (ALT), alkaline phosphatase (ALP), total cholesterol (TCHO), thyroglobulin (TG), and total protein (TP) in the serum were detected by a biochemical analyzer. Besides, the tissues of tongue, esophagus, small intestine, liver, and kidney were taken for H&E staining, and the morphological changes were observed.

### Statistical analysis

All data are expressed as mean ± SEM, and are plotted by GraphPad Prism 5.0.1 scientific statistical mapping software. Statistically significant differences among the groups were analyzed by one-way ANOVA, followed by Bonferroni’s post hoc tests. *p* < 0.05 was considered to be statistically significant, and the levels of differences were denoted in the caption of the corresponding figure.

## Results

### Decreased expression of COX-2, β-catenin, and MMP-9 proteins in the boiling water-burned wound tissue under ShengFu oil treatment

**FIGURE 1:**
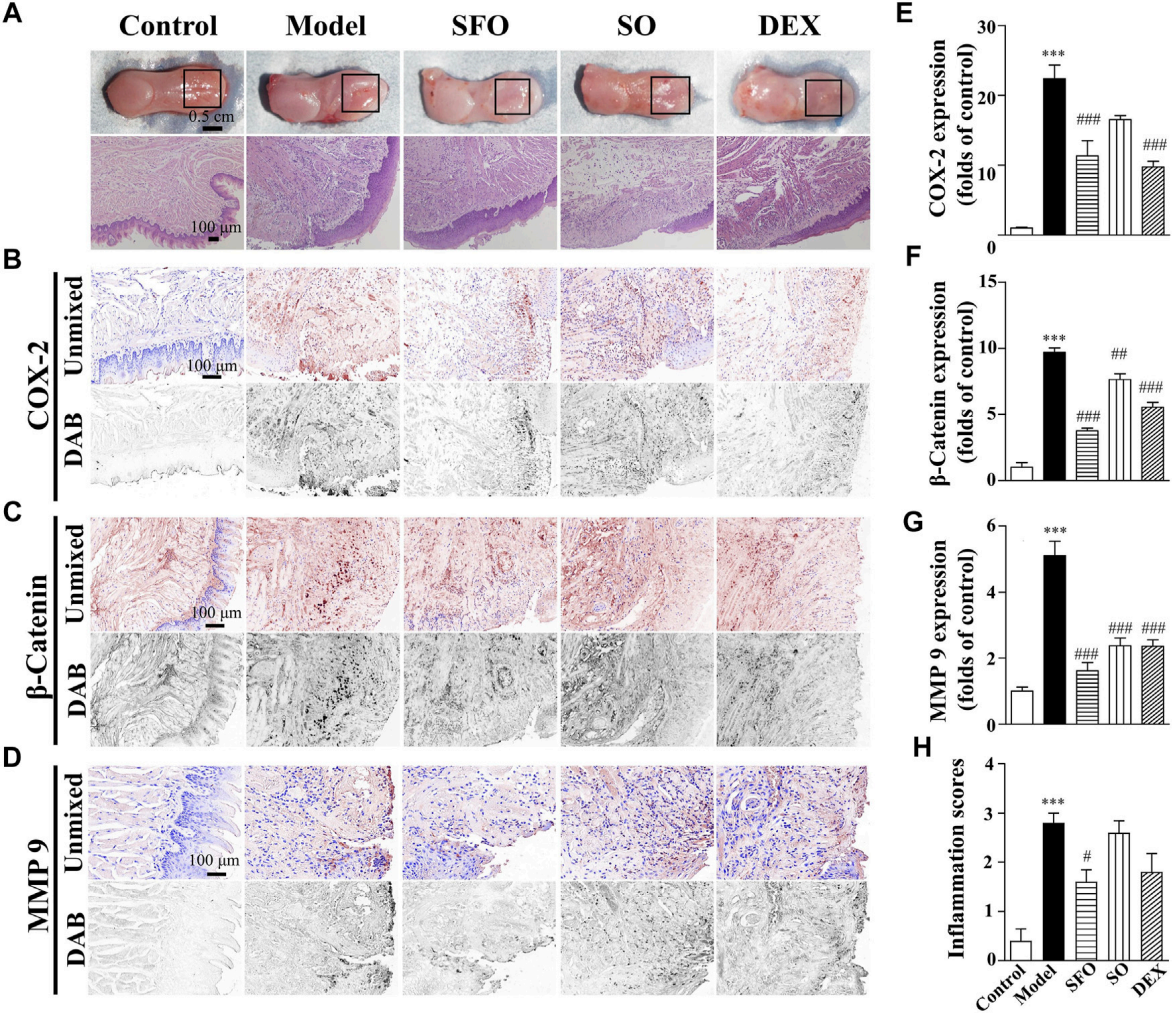
Wound healing acceleration by ShengFu oil in tongue of boiling water-burned mice. **(A)** General view of the tongue samples, and H&E staining of tongue sections in control, model, ShengFu oil (SFO), dexamethasone (DEX), and sesame oil (SO) groups. Representative **(B–D)** and summary **(E–G)** of histochemistry analysis of expression of COX-2, *β*-catenin and MMP-9, respectively. **(H)** The inflammation scores of the tongue samples in the H&E-stained images. Scale bar, 100 µm. Compared with the control group, ∗∗∗, *p* < 0.001. Compared with the model group, #, *p* < 0.05, ##, *p* < 0.01, ###, *p* < 0.001.

Our previous study reported the positive effects of ShengFu oil on scalded rabbits with Ⅲ degree scald (Chen et al., 2017). In the current study (Figure 1), we observed that the burn by boiling water seriously damaged the tongue tissue in the model group. The burns of model mice were intensely painful to the touch with brisk capillary refill. The burn wound was pink to red with punctate bleeding, swollen and blistered. The wound was still confined to the epidermis of oral mucosal surface, and it had a shallower depth than the acid- and alkali-burned wounds. H&E staining of the wounds in the model group demonstrated tissue swelling and absence of the epithelial cell layer. Meanwhile, aggregation of inflammatory cells and erythrocytes increased significantly in the lamina propria and muscular layer. On the other hand, histopathological sections illustrated swelling and thickening of the epithelial layer, disappearance of the papillae, increase in the inflammatory cells, and the disappearance of the orderly structure of the basal cells. The wound surface of mice in the sesame oil group was similar. Contrastingly, in the dexamethasone group, the wound edema was alleviated, and the blood cells decreased. However, there was increased inflammatory cell infiltration in the muscular layer. After treatment with ShengFu oil, the infiltration of inflammatory cells in the wound tissue of mice decreased significantly, and there was no blood cell aggregation. Compared to the control group, the inflammation scores were significantly increased in mice exposed to boiling water (2.80 ± 0.24-fold of control). Administration of SFO (1.60 ± 0.24-fold of control) and DEX (1.80 ± 0.37-fold of control) significantly reduced inflammatory responses compared with the model group.

To further demonstrate the efficacy of ShengFu oil, we monitored changes of several key factors (including COX-2, β-catenin, and MMP-9), closely associated with inflammatory response. As depicted in the thermal burn model in Figure 1, the expression of COX-2 in the tongue wound tissue of the model group (2237 ± 195% of control, *p <* 0.001) was significantly increased compared with that in the control group (100 ± 13%). After drug treatment, the expression of COX-2 in the sesame oil group (1,652 ± 57% of control) was not different from that in the model group, while that in the ShengFu oil (1,130 ± 223% of control, *p* < 0.001) and dexamethasone (974 ± 82% of control, *p* < 0.001) groups were significantly decreased.

The levels of β-catenin protein expression in the tongue of the mice exposed to boiling water (970 ± 33% of control, *p <* 0.001) were greatly increased compared with that in the control group. Compared to the model group, the sesame oil (764 ± 45% of control, *p <* 0.01), dexamethasone (554 ± 37% of control, *p <* 0.001), and ShengFu oil (376 ± 21% of control, *p <* 0.001) significantly reduced β-catenin expression.

As one of the downstream factors of COX-2, MMP-9 is a major protease that degrades extracellular matrix and participates in tissue injury. Our results revealed that compared with the control group (100 ± 12%), the expression of MMP-9 in the tongue wound tissue was significantly increased in the model group (510 ± 44% of control, *p <* 0.001). After pharmacological treatment, there was significant difference in the expression of MMP-9 between the dexamethasone (236 ± 19% of control, *p <* 0.001) and sesame oil (237 ± 23% of control, *p <* 0.001) groups compared with the model group. However, the expression of MMP-9 in the ShengFu oil group also decreased significantly (162 ± 25% of control, *p <* 0.001).

### Decreased expression of COX-2, β-catenin, and MMP-9 proteins in the glacial acetic acid-burned wound tissue under ShengFu oil treatment

**FIGURE 2:**
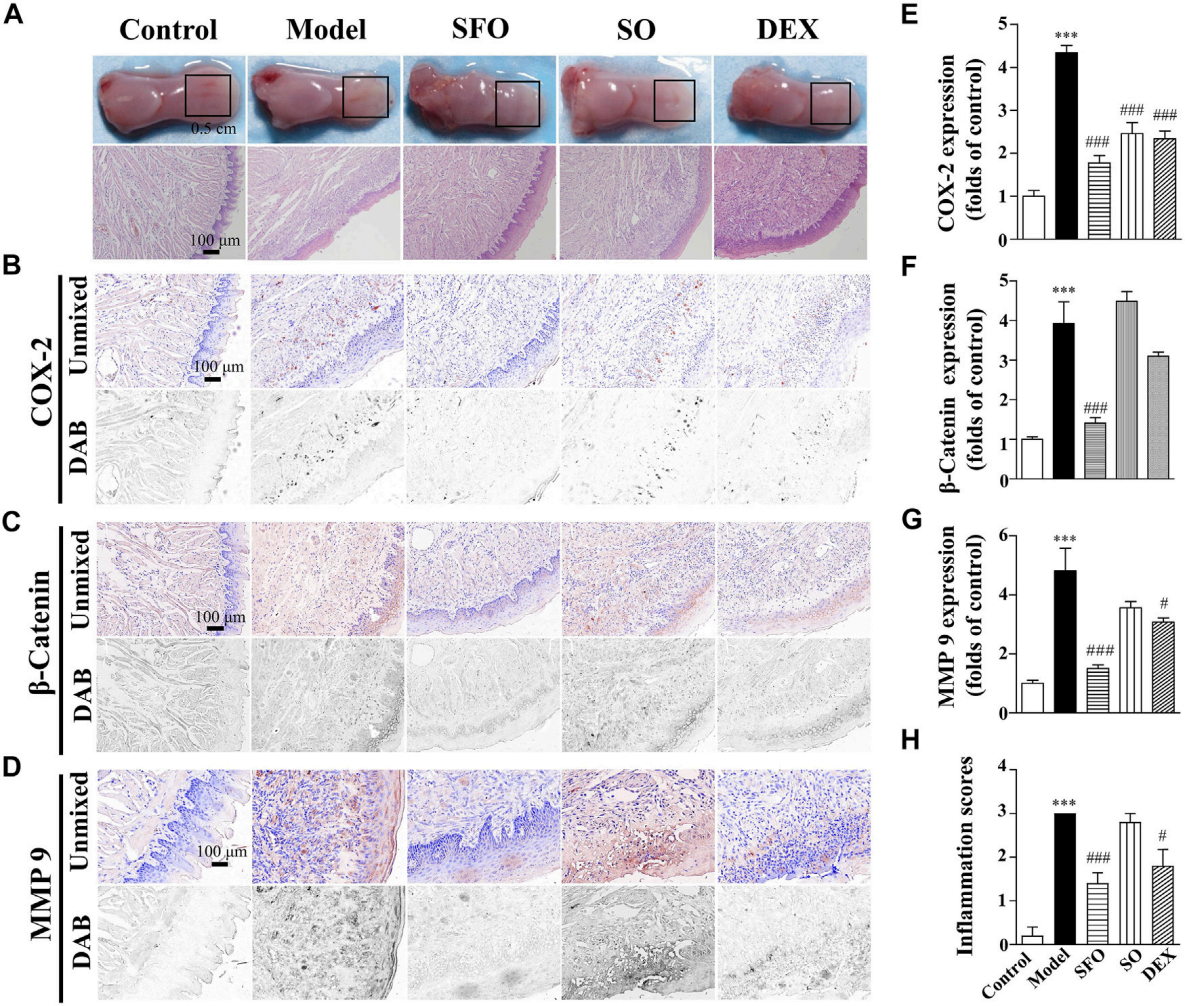
Effect and repair mechanism of ShengFu oil in tongue of glacial acetic acid-burned mice. **(A)** General view of the tongue samples, and H&E staining of tongue sections in control, model, ShengFu oil (SFO), dexamethasone (DEX), and sesame oil (SO) groups. Representative **(B–D)** and summary **(E–G)** of histochemistry analysis of expression of COX-2, *β*-catenin and MMP-9, respectively. **(H)** The inflammation scores of the tongue samples in the H&E-stained images. Scale bar, 100 µm. Compared with the control group, ∗∗∗, *p* < 0.001. Compared with the model group, #, *p* < 0.05, ###, *p* < 0.001.

Subsequently, we investigated the therapeutic effect of ShengFu oil in the tongue of glacial acetic acid-exposed mice. The burned mice were slightly painful to the touch with slow capillary refill. The injury reached the oral submucosa, accompanying slight bleeding, blisters, and swollen around. The manifestations of coagulative necrosis were noted, such as dry surface, callused wound bed, and clear boundary. As depicted in Figure 2, compared with the control group, a new keratinized layer came into being in the tongue wound tissue of the model group, while the epithelial structures, such as papillae on the lingual and taste buds, remained unseen, with the lamina propria thickened from edema. Meanwhile, there was increased inflammatory infiltration in the wound tissue. The pathological changes of tongue wounds in the sesame oil group were similar to those in the model group and inflammatory cell infiltration in the tongue wound of the dexamethasone-group mice. However, after treatment with ShengFu oil, oral epithelium of the tongue wound consisted of well-ordered stratified cells and tight intercellular junctions, the lamina propria edema vanished, and there was no apparent inflammatory cell infiltration in the tissue. Compared to the control group, the inflammation scores were significantly increased in mice exposed to acid (3.00-fold of control). Administration of SFO (1.60 ± 0.24-fold of control) and DEX (1.80 ± 0.37-fold of control) significantly reduced inflammatory responses compared with the model group.

As illustrated in Figure 2, COX-2 expression in the tongue wound tissue of the mice exposed to glacial acetic acid rose dramatically when compared to that in the control group. Compared with the model group, COX-2 expression in tongue wound of mice in the dexamethasone (246 ± 25% of control, *p* < 0.001) and sesame oil (234 ± 18% of control, *p* < 0.001) groups were lower than that in the model group. Additionally, the expression of COX-2 in the ShengFu oil group (178 ± 17% of control, *p* < 0.001) decreased significantly.

Meanwhile, we detected the effect of β-catenin expression. Our result showed that compared with the control group (100 ± 7%), β-catenin expression in the tongue wound of the model group was significantly increased (392 ± 55% of control, *p* < 0.001). Compared with the model group, β-catenin expression in the tongue wound of mice in the ShengFu oil group (141 ± 14% of control, *p* < 0.001) was significantly decreased. However, there was no significant difference in β-catenin expression between the sesame oil (448 ± 25% of control) and dexamethasone (310 ± 11% of control) groups and the model group.

We observed that MMP-9 expression in the tongue wound of the model group (481 ± 77% of control, *p* < 0.001) was significantly higher than that of the control group (100 ± 11%). There was no significant difference in MMP-9 expression between the sesame oil group (356 ± 21% of control, *p <* 0.05) and the model group. However, MMP-9 expression in the ShengFu oil (150 ± 12% of control, *p* < 0.001) and dexamethasone (307 ± 14% of control) groups decreased significantly.

### Decreased expression of COX-2, β-catenin, and MMP-9 proteins in the sodium hydroxide-burned wound tissue under ShengFu oil treatment

Next, we investigated the therapeutic effect of ShengFu oil in the tongue of sodium hydroxide-exposed mice. The sodium hydroxide-burned wound was more severe than boiling water- and acid-burned wounds, and the damage was visible by the naked eye as far as the muscular layer. There was almost no pain perception when pressed with no capillary refill. A circumscribed yellowish-brown blister with sloughing white lesion of the mucosa adjacent to it was noted. Compared with the control group, the wound epithelium of the model group exfoliated to form ulcers, epithelial layer cells around the injured tissue swelled, basal layer structure was disordered, and there was increased infiltration of inflammatory and blood cells in the muscle layer. The histopathological changes of the wound in the sesame oil and dexamethasone groups were similar to those in the model group, with increased infiltration with inflammatory and red blood cells at the injury site. However, the inflammatory infiltration in the ShengFu oil group was significantly less than that in the model group; further, there was no blood cell aggregation in the tissue, and the tissue structure was intact. Compared to the control group, the inflammation scores were significantly increased in mice exposed to alkali (3.00-fold of control). Administration of SFO (1.40 ± 0.24-fold of control) and DEX (2.00 ± 0.32-fold of control) significantly reduced inflammatory responses compared with the model group.

**FIGURE 3:**
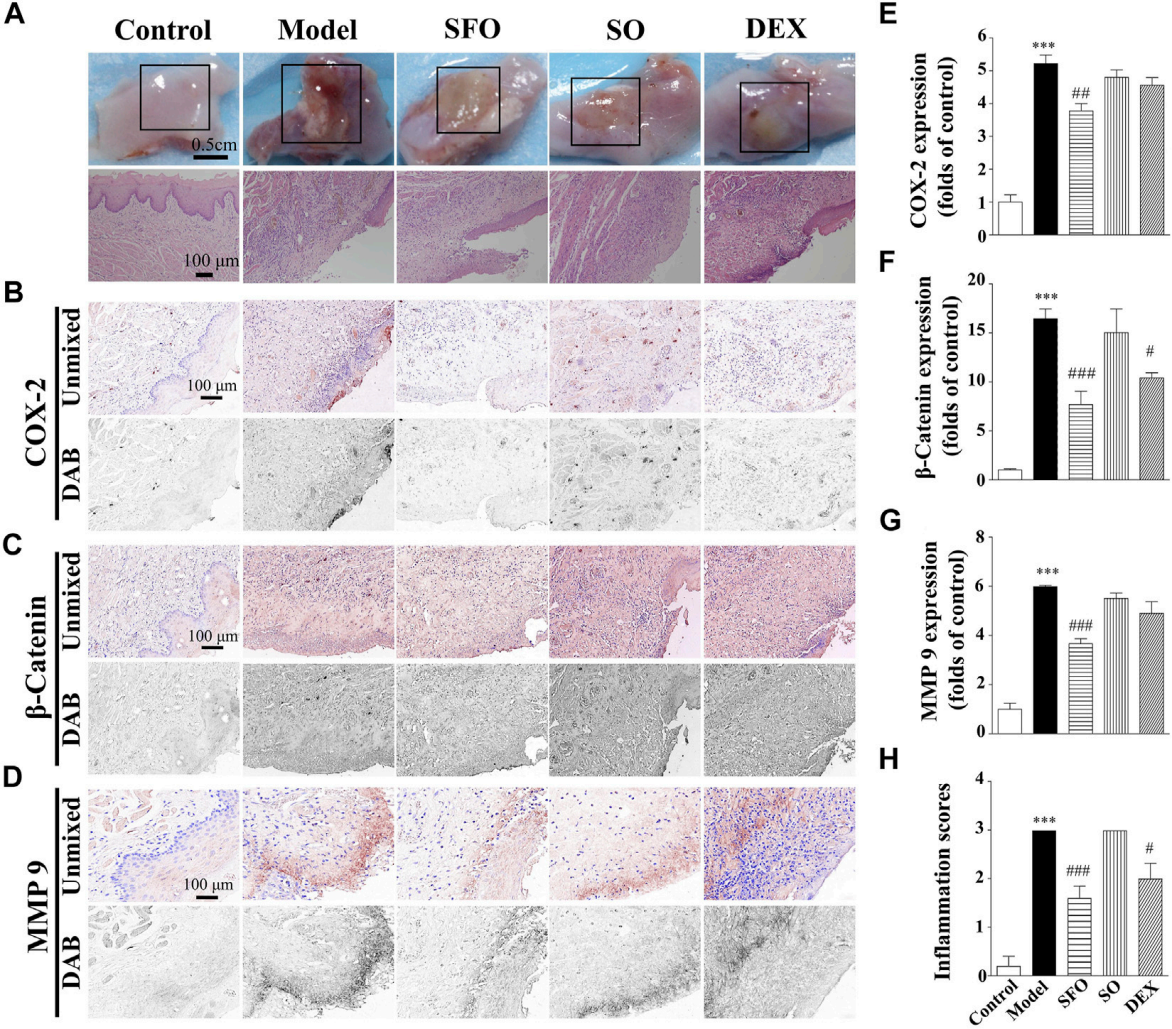
Decreased expression of COX-2, β-catenin and MMP-9 protein in the sodium hydroxide-burned wound tongue tissue of mice under ShengFu oil treatment. **(A)** General view of the tongue samples, and H&E staining of tongue sections in control, model, ShengFu oil (SFO), dexamethasone (DEX), and sesame oil (SO) groups. Representative **(B–D)** and summary **(E–G)** of histochemistry analysis of expression of COX-2, *β*-catenin and MMP-9, respectively. **(H)** The inflammation scores of the oral mucosa samples in the H&E-stained images. Scale bar, 100 µm. Compared with the control group, ∗∗∗, *p* < 0.001. Compared with the model group, #, *p* < 0.05, ##, *p* < 0.01, ###, *p* < 0.001.

As shown in Figure 3, compared with the control group (100 ± 22%), COX-2 expression in the model group was significantly increased (522 ± 26% of control, *p* < 0.001). However, no significant difference in COX-2 expression was observed in the sesame oil (480 ± 23% of control) or dexamethasone (456 ± 23% of control) groups when compared with the model group. In contrast, COX-2 expression in the ShengFu oil group (377 ± 23% of control, *p* < 0.01) decreased significantly. The level of *β*-catenin protein expression in the tongue of the mice exposed to sodium hydroxide was greatly increased compared with that in the control group. Compared with the control group (100 ± 13%), β-catenin expression in the model group was significantly increased (1,646 ± 98% of control, *p* < 0.001). Compared with the model group, only β-catenin expression in the ShengFu oil group (769 ± 135% of control, *p* < 0.001) decreased significantly. Meanwhile, we also detected MMP-9 expression in sodium hydroxide-burned mice. The multispectral quantitative results demonstrated a significantly higher expression of MMP-9 in the wound tissue of the model group (599 ± 5% of control, *p* < 0.001) compared to the control group (100 ± 25%). Compared with the model group, only the expression of MMP-9 in the ShengFu oil group (367 ± 21% of control, *p* < 0.001) decreased significantly.

### Repair of oral burns caused by sodium hydroxide could be associated with the promoting effect of Frankincense

**FIGURE 4:**
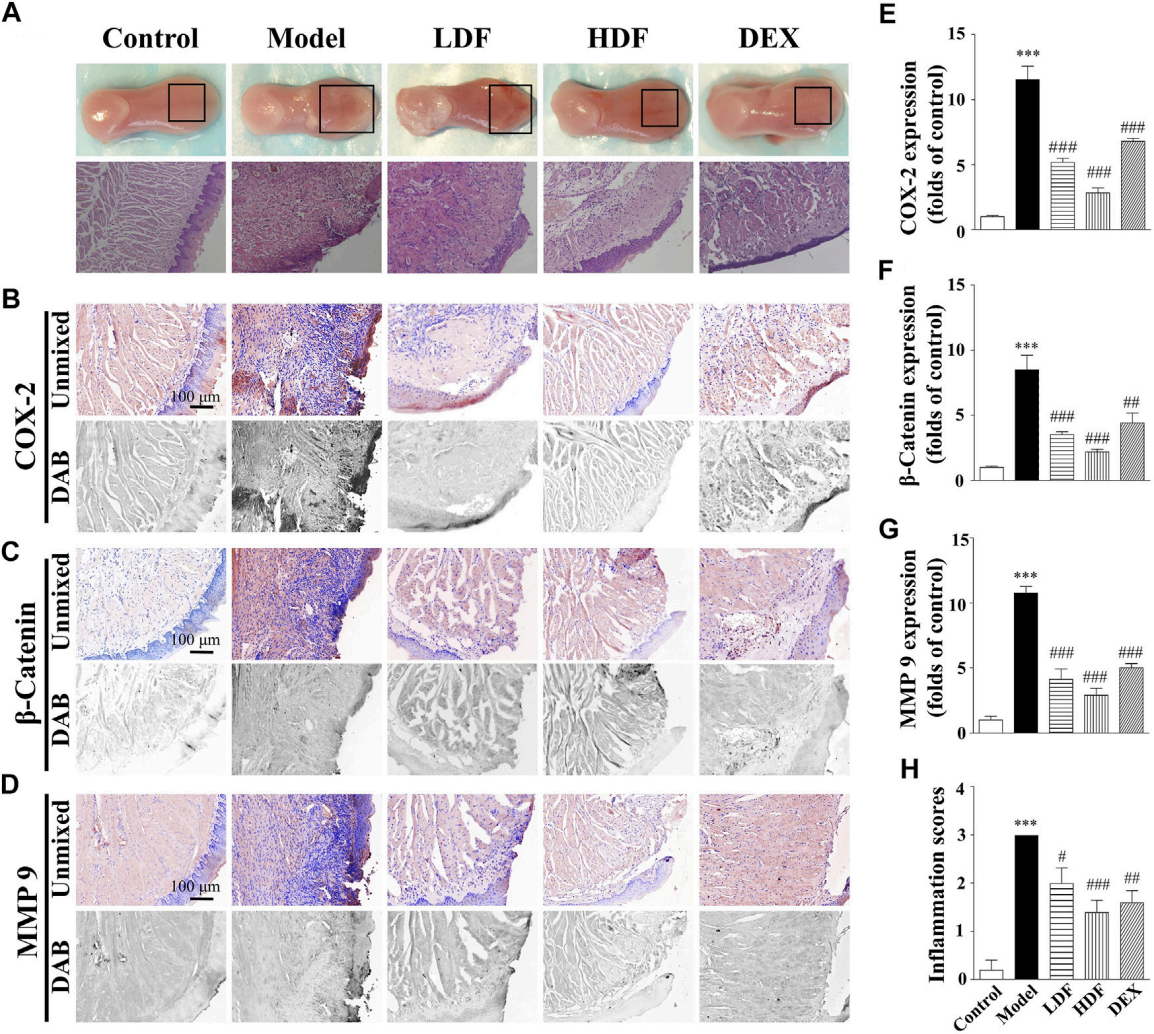
Wound healing acceleration of Frankincense for sodium hydroxide-caused oral burns in mice. **(A)** General view of the tongue samples, and H&E;staining of tongue sections in control, model, Frankincense (LDF and HDF are denoted low- and high-dose groups, respectively), and dexamethasone (DEX) groups, respectively. Representative **(B–D)** and summary **(E–G)** of histochemistry analysis of expression of COX-2, *β*-catenin and MMP-9, respectively **(H)** The inflammation scores of the tongue samples in the H&E-stained images. Scale bar, 100 µm. Compared with the control group, ∗∗∗, *p* < 0.001. Compared with the model group, #, *p* < 0.05, ##, *p* < 0.01, ###, *p* < 0.001.

In accordance with our previous study, Frankincense is one of the important components of ShengFu oil. As has been mentioned above, alkali-caused burns are the most severe of the three chemical burns and the most damaging to the oral mucosa. Therefore, we investigated the therapeutic effect of Frankincense extract in the tongue of sodium hydroxide-exposed mice. As shown in Figure 4, compared with the control group, the extracellular matrix of the wound tissue was destroyed and the epithelial structure exfoliated. The epithelial layer cells around the injured tissue swelled and basal layer structure was disordered. Moreover, there was infiltration of inflammatory and blood cells in the muscular layer of the tissue; the lamina propria was thickened. The histopathological changes of the wound in the low-dose Frankincense extract and dexamethasone groups were superior to those in the model group, but not good as those in the high-dose Frankincense extract group. The inflammatory infiltration in the high-dose Frankincense extract group was significantly less than that in the other groups. There was no blood cell aggregation in the tissue, and the basal layer cells around the damaged tissue were observed to gradually migrate to the injury site and form new epithelium. Compared to the control group, the inflammation scores were significantly increased in mice exposed to alkali (3.00-fold of control). Administration of LDF (2.00 ± 0.32-fold of control), HDF (1.40 ± 0.24-fold of control) and DEX (1.60 ± 0.24-fold of control) significantly reduced inflammatory responses compared with the model group.

As shown in Figure 4, the COX-2 expression in the tongue wound tissue of the alkali-burn model group (1,153 ± 104% of control, *p <* 0.001) was significantly increased compared with that in the control group (100 ± 11%). After treatment, COX-2 expression in the drug-given groups were obviously different from that in the model group, while those in the low-dose Frankincense extract (517 ± 30% of control, *p <* 0.001), high-dose Frankincense extract (283 ± 37% of control, *p <* 0.001), and dexamethasone (679 ± 25% of control, *p <* 0.001) groups were significantly decreased.

Compared with the control group (100 ± 10%), β-catenin expression in the tongue wound tissue of mice in the model group (850 ± 11% of control, *p <* 0.001) was significantly increased. Compared with the model group, β-catenin expression was significantly decreased in the low-dose Frankincense extract (353 ± 22% of control, *p <* 0.001), high-dose Frankincense extract (219 ± 21% of control, *p <* 0.001), and dexamethasone (442 ± 76% of control, *p <* 0.01) groups. The effect of the high-dose Frankincense extract group was the best.

Compared with the control group (100 ± 29%), MMP-9 expression in the tongue wound tissue of the model group (1,077 ± 48% of control, *p* < 0.001) was significantly increased. Compared with the model group, MMP-9 expression in the low-dose Frankincense extract (412 ± 81 of control, *p* < 0.001), high-dose Frankincense extract (290 ± 55 of control, *p* < 0.001), and dexamethasone (500 ± 34% of control, *p* < 0.001) groups were significantly down-regulated. The therapeutic effect of Frankincense extract was proportional to the dosage within a certain range.

### Repairs of oral burns caused by sodium hydroxide could be associated with the promoting effect of α-pinene, linalool, and 1-octanol

**FIGURE 5:**
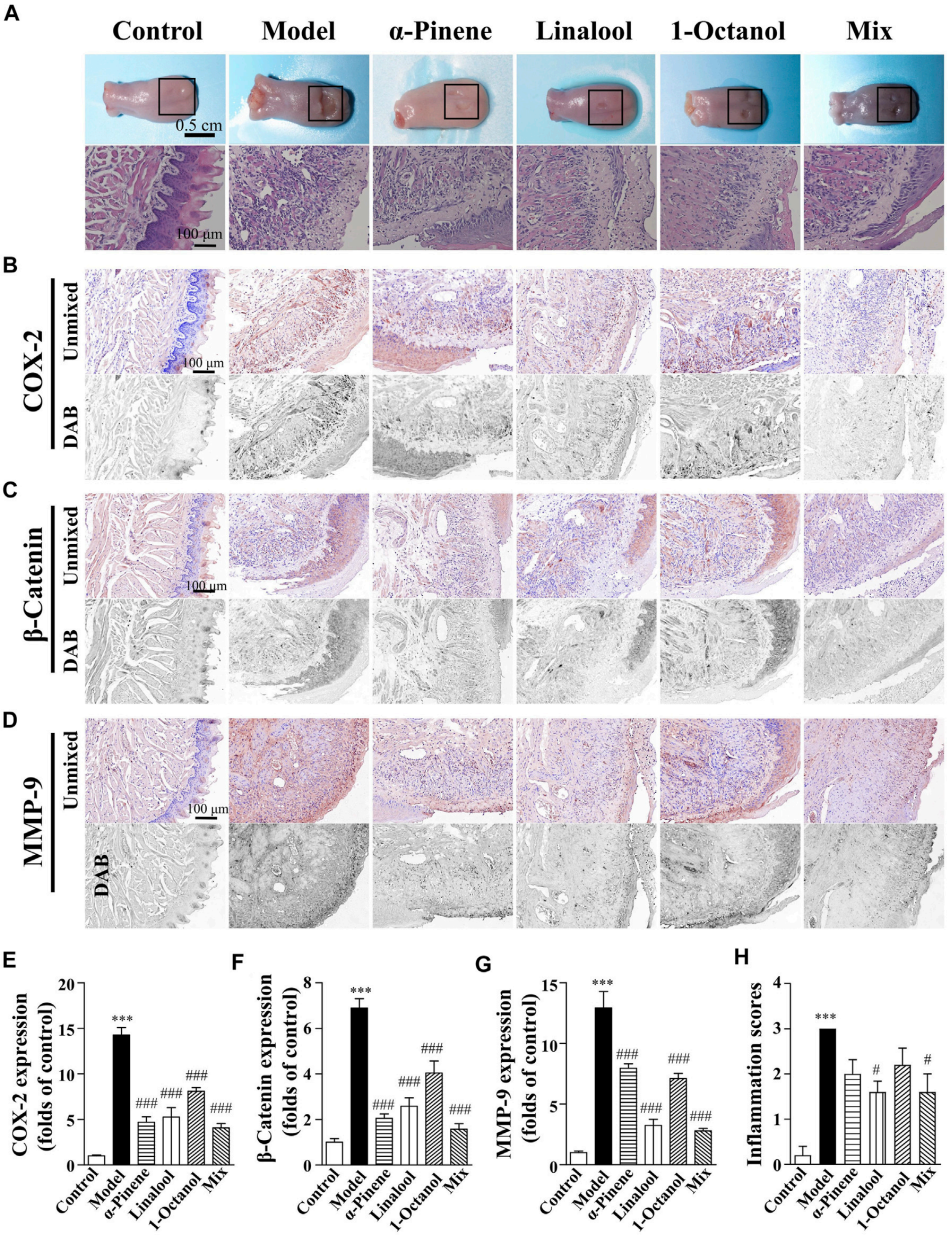
Wound healing acceleration of α-pinene, linalool and 1-octanol for sodium hydroxide-caused oral burns in mice. **(A)** General view of the tongue samples, and H&E staining of tongue sections in control, model, ShengFu oil (SFO), dexamethasone (DEX) and sesame oil (SO) groups, respectively. Representative **(B–D)** and summary **(E–G)** of histochemistry analysis of expression of COX-2, *β*-catenin and MMP-9, respectively. **(H)** The inflammation scores of the tongue samples in the H&E-stained images. Scale bar, 100 µm. Compared with the control group, ∗∗∗, *p* < 0.001. Compared with the model group, #, *p* < 0.05, ###, *p* < 0.001.

According to our previous study, Frankincense is one of the important components of ShengFu oil. Its active components α-pinene, linalool, and 1-octanol and their mixture have the effect of detumescence and analgesia. In this study, we investigated the therapeutic effects of α-pinene, linalool, and 1-octanol in the tongues of sodium hydroxide-exposed mice. As shown in Figure 5, compared with the control group, the extracellular matrix of the wound tissue was destroyed and the epithelial structure was disintegrated in the model group. Moreover, the lamina propria was thickened with inflammatory cell infiltration, and there was inflammatory and blood cells infiltration in the muscular layer of the tissue. In contrast, the epithelial structure of the wound partially disappeared, and the edema of the lamina propria was alleviated in drug-administered groups. Furthermore, there was inflammatory cell infiltration in the muscle layer, but it was significantly less than that in the model group. Moreover, the basal layer cells around the injured tissue gradually migrated to the injured site, forming new epithelium. Compared to the control group, the inflammation scores were significantly increased in mice exposed to alkali (3.00-fold of control). Administration of α-pinene (2.00 ± 0.32-fold of control), linalool (1.60 ± 0.24-fold of control)), and 1-octanol (2.20 ± 0.37-fold of control) and their mixture (1.60 ± 0.40-fold of control) significantly reduced inflammatory responses compared with the model group.

Compared with the control group (100 ± 9%), COX-2 expression in the tongue wound tissue of the model group (1,426 ± 82% of control, *p* < 0.001) increased significantly. Compared with the model group, COX-2 expression was significantly decreased in the linalool (526 ± 103% of control, *p* < 0.001), 1-octanol (808 ± 42% of control, *p* < 0.001), and mixed (408 ± 46% of control, *p* < 0.001) groups. The expression of COX-2 in tongue wound tissue of mice in α-pinene group (469 ± 59% of control, *p* < 0.001) also showed a downward trend.

Compared with the control group (100 ± 16%), β-catenin expression in the tongue wound tissue of mice in the model group (689 ± 41% of control, *p* < 0.001) was significantly increased. Compared with the model group, β-catenin expression was significantly decreased in the α-pinene (205 ± 19% of control, *p* < 0.001), linalool (258 ± 37% of control, *p* < 0.001), 1-octanol (404 ± 53% of control, *p* < 0.001), and mixed (158 ± 24% of control, *p* < 0.001) groups. The effect of mixed group was the best.

Compared with the control group, MMP-9 expression in the tongue wound tissue of the model group was significantly increased (1,293 ± 135% of control, *p* < 0.001). Compared with the model group, MMP-9 expression in the α-pinene (795 ± 38 of control, *p* < 0.001), linalool (324 ± 51 of control, *p* < 0.001), 1-octanol (710 ± 43% of control, *p* < 0.001), and mixed (279 ± 20% of control, *p* < 0.001) groups were significantly down-regulated.

### Repeated dose 30-days oral toxicity study of ShengFu oil in mice

**FIGURE 6:**
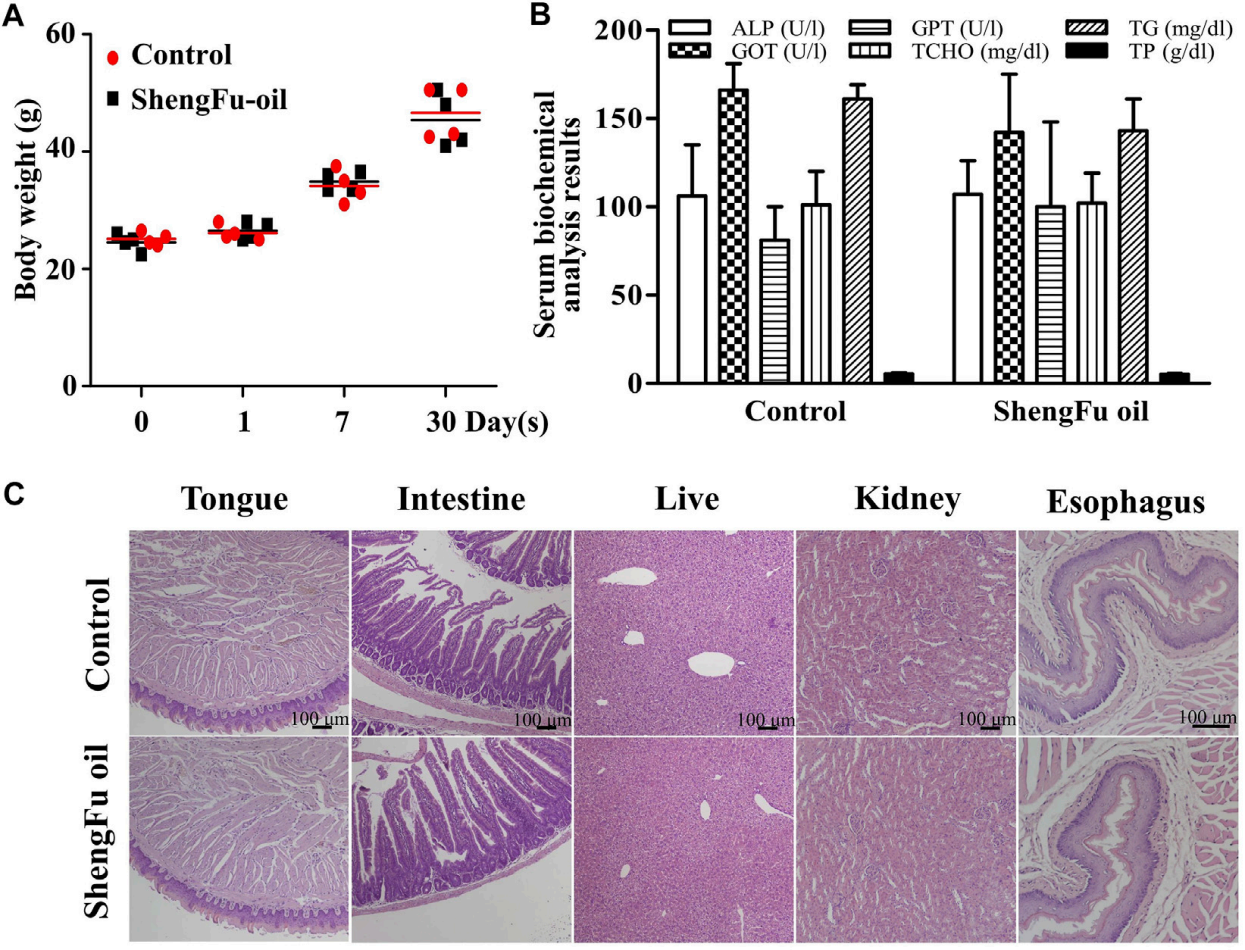
No harmful effects of ShengFu oil on normal organs including tongue, esophagus, small intestine, liver, and kidney of mice. **(A)** Body weight gain in different groups during the experiment. **(B)** Serum biochemical analysis results of a 30-days safety assessment in young mice orally exposed to ShengFu oil (SFO). The changes in serum AST, ALT, ALP, TCHO, TG, and TP aspartate aminotransferase (AST), alanine aminotransferase (ALT), alkaline phosphatase (ALP), total cholesterol (TCHO), thyroglobulin (TG), and total protein (TP) levels directly or indirectly reflect the damage. Data were presented as mean ± SEM. **(C)** H&E staining of tissue samples from tongue, esophagus, small intestine, liver and kidney in control and ShengFu oil groups.

As shown in Figure 6A, after 30 days of administration of ShengFu oil, the body weight of mice showed no significant differences compared with the control group. Serum biochemical analysis results (Figure 6B) of a 30-days safety assessment in young mice orally exposed to ShengFu oil showed that there was no significant difference in the serum levels of AST, ALT, ALP, TCHO, TG, and TP between the ShengFu oil and normal groups. H&E staining results (Figure 6C) showed that there was no difference in the tongue, esophagus, small intestne, liver, and kidney between the ShengFu oil and normal groups.

## Discussion

In the absence of a specific clinical treatment guide for the chemical burn injuries of the oral mucosa, oral burn treatment can only be learnt from the treatment modalities of the skin burns because the differences between the skin and oral mucosa are always ignored (Allorto et al., 2018). Nevertheless, oral mucosa, which is significantly less protective against external stimuli than the skin tissue, is different from the skin (Nikoloudaki et al., 2020). Thus, we need a treatment different from that for the skin burns. Although its general treatment often follows the principles for thermal burns (Nguyen et al., 2021), chemical burns differ from thermal burns because of differences in the mechanisms involved in the pathophysiology of the mucosal damage (Choi et al., 2009). Therefore, it is necessary for us to illustrate integrally the regulatory mechanism of oral chemical burn healing and elucidate the burn wound healing effect of ShengFu oil on the oral mucosa. According to the etiology, oral chemical burns can be divided into acid- and alkali-induced burns (Narkutė and Žilinskaitė, 2019). Acids with pH value less than two cause coagulative necrosis of the oral mucosal epithelial cells and leave eschar on the wound surface (Akelma and Karahan, 2019). Eschar limits penetration of harmful chemical substances from the epidermis into the deeper submucosa, which can reduce the degree of injury. Unlike acid-induced damage, alkalis can capture hydrogen ions from protonated amines and carboxyl groups; hence, lye, with a pH value greater than 11.5, can cause severe tissue damage through liquefaction necrosis (Lupa et al., 2009; Contini and Scarpignato, 2013). Moreover, alkalis can also penetrate the mucosal matrix and destroy proteoglycans and collagen, leading to saponification of fatty acids in the cell membrane, which further aggravates tissue damage (VanHoy et al., 2022). Although chemical burns have been extensively studied, the molecular determinants of chemical substance-induced oral chemical burns and inflammatory cytokine-mediated repair of oral chemical burns remain scarcely explored. Our previous study reported Frankincense to be one of the main components of ShengFu oil against inflammation (Jia et al., 2013). Its active components α-pinene, linalool, and 1-octanol and their mixture have the effect of detumescence and analgesia (Li et al., 2016; Chen et al., 2017). However, the efficacy of Frankincense and its three active ingredients for the treatment of oral chemical burns has not been demonstrated by molecular mechanisms. To find a therapeutic modality for oral chemical burns, it is pertinent to study the molecular aspects of oral chemical burns. In this study, treatment effects of ShengFu oil and its significant ingredient Frankincense on oral burns was studied by establishing the models of oral burn caused by heat, acid, and alkali.

Wound healing is influenced by many factors that mainly rely on the proliferation and migration of fibroblasts (Dekoninck and Blanpain, 2019). Some proteins, such as β-catenin and COX-2, are closely associated with wound healing (Li et al., 2016; Chen et al., 2017). Inflammation is an integrative process driven by a number of mediators which regulate specific aspects of the inflammatory response (Fioranelli et al., 2021). Under normal conditions, inflammatory reactions will promote the proliferation of adult fibroblasts (Carre et al., 2010), up-regulate the production of β-catenin (Schunk et al., 2021), COX-2, and the secretion of prostaglandins (Enzerink et al., 2010), activate vascular endothelial growth factors (VEGFs), and promote wound healing by facilitating wound angiogenesis and accelerating blood supply (de Oliveira et al., 2014). Nevertheless, overexpression of β-catenin has adverse effects including keloids and hypertrophic scars on wound healing because it abnormally induces continuous activation of the Wnt/β-catenin signaling pathway (Chua et al., 2011; Clevers and Nusse, 2012; Guo et al., 2012). The constitutive activation of β-catenin and Wnt signaling can lead to over-expression of VEGF (Nasser et al., 2021). It has been reported that VEGF-1 is regulated by NF-κB in LPS-stimulated human primary macrophages, and control the transcriptions of COX-2 and pro-inflammatory cytokines (VEGF1, TNFα; Cho et al., 2011).

COX-2 may up-regulate the TGFβ1 expression. The COX-2 inhibitor should also have the effect of enhancing the cell proliferation through the suppression of TGFβ1 expression (Baek et al., 2001). It is reported that TGF-β induced the upregulation of Wnt/β-catenin signalling in hypertrophic scar and keloid fibroblasts. The role of TGFβ in impairing ECM remodeling and antiangiogenic resistance (Baek et al., 2001) has been demonstrated in various fibrotic models (Semkova and Hsuan, 2021).

As one of the downstream factors of COX-2, MMP-9 is a major protease that degrades extracellular matrix (ECM) and participates in tissue injury (Bandara and MacNaughton, 2021). In the chemical burn wound, excessive activation of MMP-9 leads to the breakdown of local ECM, and cell migration is impaired without the scaffold provided by ECM (Lan et al., 2021). Therefore, high expression of MMP-9 is indicative of poor wound healing (Zhou et al., 2018), which increases the risk of further infection, leading to prolonged inflammation, low levels of growth factors at the wound site, and further delay the wound healing (Banerjee et al., 2021). As a result, the reduction of β-catenin, COX-2 and MMP-9 can improve wound healing.

Our previous studies showed that while inhibiting the production of COX-2, Frankincense can reduce the activity of MMP-9, inhibit the destruction of collagen matrix, prevent collagen degradation, and thus inhibit inflammatory reaction (Li et al., 2016). Undoubtedly, it demonstrated the synergistic participation of MMP-9 and COX-2 in tissue damage. Therefore, we quantitatively analyzed the expression of COX-2, β-catenin, and MMP-9 during healing of oral chemical burn mucosa model as a judgment standard to illustrate integrally the regulatory mechanism of oral chemical burn healing. Firstly, general observation illustrated that the thermal injury of tongue mucosa was red, swollen, and bleeding, accompanied by blisters on the wound surface. The acid burn tongue tissue wound was dry, the wound edge was clear, and there were no blisters. Furthermore, the alkali burn oral mucosa wound was sticky and slippery, with small blisters, and the wound was deeper than others. We hypothesized that the differences among the three kinds of wounds were due to the different histopathological changes caused by heat, acid, and alkali. In thermal burns, it is the latent heat high-temperature liquid release, which comes in contact with the mucosa, that builds an irreversible protein cross-linking reaction in the tissue and damages the tissue (Frantz and Byers, 2011). In acid burn, glacial acetic acid causes dehydration and protein coagulation of tongue tissue, resulting in local mucosal coagulation necrosis (Friedenwald et al., 1946; Bizrah et al., 2019). In alkali burns, sodium hydroxide dehydrates oral mucosal tissue cells and saponifies fat, and at the same time dissolves proteins, resulting in mucosal liquefaction necrosis (Atug et al., 2009; Mutoji et al., 2021). As a result, alkali injuries cause more damage to the mucosa than acid or thermal injuries (Wagoner, 1997).

In this study, we found that after the treatment with ShengFu oil, all three kinds of burn wounds improved, the exudation of wound tissue fluid significantly reduced, there was no bleeding, and the edema was significantly reduced. Next, we further explored the anti-inflammatory efficacy of Frankincense in oral chemical burned mucosa. The main components α-pinene, linalool, and n-octanol of Frankincense also reduced wound inflammation, promoted the migration of surrounding basal cells to the wound, promoted the proliferation of fibroblasts and endothelial cells, and accelerated wound repair. In previous studies, we demonstrated that ShengFu oil can enhance wound healing by regulating the expression of β-catenin, and COX-2 (Chen et al., 2017). In addition, our results showed that the high expression of β-catenin, COX-2 and MMP-9 in oral thermal, acid, and alkali burns were downregulated after treatment with ShengFu oil.

Therefore, we proposed a hypothesis that after oral chemical burns, excessive accumulation of inflammatory leukocytes in the wound tissues releases cytokines, induces the expression of COX-2, activates β-catenin, and forms an inflammatory loop with β-catenin to participate in the maintenance of inflammatory response, in which PGE_2_, EP_2_, TGF-β1, and VEGF-1 played as signal molecules (Li et al., 2018). At the same time, COX-2 can also induce the expression of MMP-9, create the COX-2/MMP-9 axis (Khan et al., 2004) via regulation of TGFβ-1 and TNF-α, promote the disintegration of ECM at the injury site, and finally make it difficult to form new tissues in the wound (Okamoto et al., 1997). Certainly, the three active components of ShengFu oil and the ingredients in Frankincense have antibacterial activity, which can reduce the risk of wound bacterial infection (Fujiwara et al., 2020), inhibit β-catenin/COX-2/MMP-9 inflammatory signal pathway, reduce wound inflammation, promote the migration of fibroblasts and epidermal cells in the matrix around the wound to the wound and accelerate the regeneration of epithelial cells and wound healing.

## Conclusion

It has been confirmed that ShengFu oil has good therapeutic effects on both acid- and alkali-induced chemical burns in oral mucosa and thermal burn caused by heat. Meanwhile, it has been clarified that the active ingredient in ShengFu oil and its main component Frankincense can reduce inflammation and accelerate wound repair by regulating the expression of β-catenin, COX-2, and MMP-9.

## Data Availability

The original contributions presented in the study are included in the article/Supplementary Material, further inquiries can be directed to the corresponding authors.
