# Supplementary material for: Enhanced healing of oral chemical burn by inhibiting inflammatory factors with an oral administration of shengFu oil
Source: Front Pharmacol. 2022 Aug 11;13:913098. doi: 10.3389/fphar.2022.913098 (PMC9403317; doi:10.3389/fphar.2022.913098)
Supplement: Supplementary file 1 [file DataSheet1.DOC]

Supplementary Material

Enhanced healing of oral chemical burn by inhibiting inflammatory factors with an oral administration of ShengFu oil

Yin Xin^1^, Jing Hong^1^, He-Bin Tang^1^, *, Min Liu^2^, *, Yu-Sang Li^1^, *

* **Correspondence:**

Yu-Sang Li: liys2006@mail.scuec.edu.cn, tel/0086-27-67841196;

Min Liu: [83792370@qq.com,](mailto:83792370@qq.com;) tel/0086-15927186600

He-Bin Tang: hbtang2006@mail.scuec.edu.cn, tel/0086-27-67842332;

# GC-MS analysis of ShengFu oil and **Frankincense oil extracts**

The chemical components of ShengFu oil and Frankincense oil extracts were detected by GC-MS on a Thermo Scientiﬁc TRACE 1300 GC Ultra system equipped with a TR-35MS capillary column (30 m×0.25 mm×0.25 μm) coupled to an ISQ system in electron ionization mode, respectively. The initial oven temperature was 50°C. Samples were maintained at 50 °C for 1 min, and the temperature was increased at a rate of 5°C/min to 200°C, where it was held for 2 min. The injector and transfer line temperatures were both 290°C.The injection volume was 1 μL. Ions were generated by a 70 eV electron beam at an ionization current of 50 μA and an ion source temperature of 250°C. The mass spectra were recorded in full scan mode (m/z 50-500) for qualitative analysis. The characteristic chromatogram of ShengFu oil has been established by GC-MS and can be acted as the quality standard to evaluate and confirm the product. Hexanal, terpinen, terpinolene, linalool and (1S)-(+)-camphorquinone may be the main components of ShengFu oil. It should be pointed out that the exact chemical components corresponding to peaks in the spectrogram will be unriddled in future. The contents of α-pinene, linalool, and 1-octanol of Frankincense oil extracts were calculated by external standard method.

# Supplementary Figure 1

**Supplementary Figure 1. GC-MS analysis of the components of ShengFu oil.** The numbers in the graph from 1 to 5 may denote hexanal, terpinen, terpinolene, linalool and (1S)-(+)-camphorquinone, respectively.

# Supplementary Figure 2


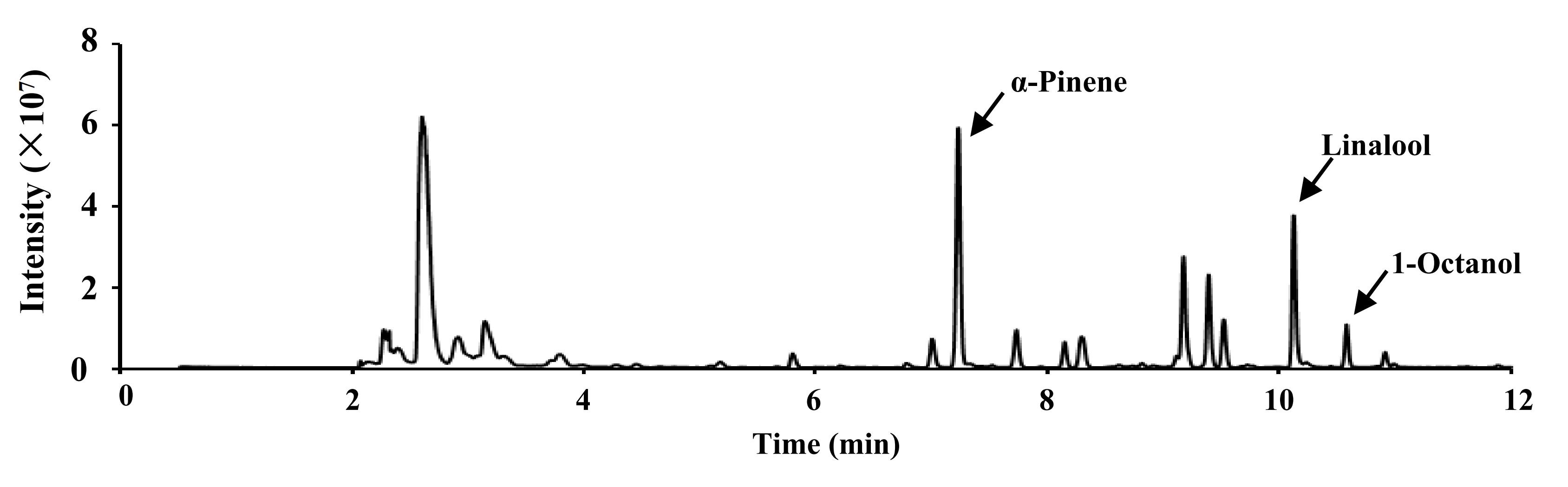


**Supplementary Figure 2. GC-MS analysis of the components of Frankincense oil extracts.**
